# Predictors of psychological distress among postnatal mothers in rural Nepal: A cross-sectional community-based study^☆^

**DOI:** 10.1016/j.jad.2013.11.018

**Published:** 2014-03-01

**Authors:** Kelly Clarke, Naomi Saville, Bhim Shrestha, Anthony Costello, Michael King, Dharma Manandhar, David Osrin, Audrey Prost

**Affiliations:** a University College London Institute for Global Health, 30 Guilford Street, London WC1N 1EH, United Kingdom; b Mother and Infant Research Activities (MIRA), P.O. Box 921, Kathmandu, Nepal; c Mental Health Sciences Unit, University College London, United Kingdom

**Keywords:** Postnatal psychological distress, Postnatal depression, Common mental disorder, Nepal, Maternal mental health, Rural health

## Abstract

**Background:**

Perinatal common mental disorders are a major cause of disability among women and have consequences for children's growth and development. We aimed to identify factors associated with psychological distress, a proxy for common mental disorders, among mothers in rural Dhanusha, Nepal.

**Methods:**

We used data from 9078 mothers who were screened for distress using the 12-item General Health Questionnaire (GHQ-12) around six weeks after delivery. We assessed the association between GHQ-12 score and socioeconomic, gender-based, cultural and reproductive health factors using a hierarchical analytical framework and multilevel linear regression models.

**Results:**

Using a threshold GHQ-12 score of ≥6 to indicate caseness, the prevalence of distress was 9.8% (886/9078). Factors that predicted distress were severe food insecurity (β 2.21 (95% confidence interval 1.43, 3.40)), having a multiple birth (2.28 (1.27, 4.10)), caesarean section (1.70 (0.29, 2.24)), perinatal health problems (1.58 (1.23, 2.02)), no schooling (1.37 (1.08, 1.73)), fewer assets (1.33 (1.10, 1.60)), five or more children (1.33 (1.09, 1.61)), poor or no antenatal care (1.31 (1.15, 1.48) *p*<0.001), having never had a son (1.31 (1.14, 1.49)), not staying in the parental home in the postnatal period (1.15 (1.02, 1.30)), having a husband with no schooling (1.17 (0.96, 1.43)) and lower maternal age (0.99 (0.97, 1.00)).

**Limitations:**

The study was cross-sectional and we were therefore unable to infer causality. Because data were not collected for some established predictors, including infant death, domestic violence and history of mental illness, we could not assess their associations with distress.

**Conclusions:**

Socioeconomic disadvantage, gender inequality and poor reproductive health predict distress among mothers in Dhanusha. Maternal and child health programmes, as well as poverty-alleviation and educational interventions, may be beneficial for maternal mental health.

## 1 Introduction

Perinatal common mental disorders (PCMDs) are a major cause of disability for women and are also associated with underweight, stunting and impaired social and cognitive development in children born to mothers suffering from these disorders (Parsons et al., 2012; Surkan et al., 2011). High rates of PCMDs have been reported in community-based studies in South Asia, with estimates ranging from 12% (95% confidence interval 11, 12) to 60% (53, 66) (Nagpal et al., 2008; Prost et al., 2012). Important predictors of PCMDs in the region include infant death (Gausia et al., 2009b; Patel et al., 2002; Prost et al., 2012), socioeconomic disadvantage (Chandran et al., 2002; Ho-Yen et al., 2007; Prost et al., 2012), poor social and family support (Gausia et al., 2009a,b; Rahman et al., 2003), domestic violence, and son preference (Ali et al., 2009; Nasreen et al., 2011; Patel et al., 2002). Studies in Nepal have estimated that the prevalence of common mental disorders in the postnatal period is in the range of 3–12%, although they involved small samples in predominantly urban areas (Ho-Yen et al., 2006; Nepal et al., 1999; Regmi et al., 2002). Among mothers, a history of depression, having a husband with alcohol problems, being in a polygamous relationship, stressful life events, multiparity and smoking were associated with postnatal depression (Ho-Yen et al., 2007). One study from Nepal reported a prevalence of common mental disorders of 50% during pregnancy, but the scale used had not been validated in Nepali and the study was conducted in tertiary care (Shakya et al., 2008). The prevalence and predictors of PCMDs have not been investigated in women living in rural Nepal, where rates of neonatal mortality are higher than urban areas, 74% of women deliver at home, and 76% access antenatal care (Central Bureau of Statistics, 2011). Provision of mental health services in rural areas is minimal and involves NGO programmes with limited coverage. In this study we investigate PCMDs in a rural area in the Terai (plains) region of Nepal to identify factors associated with psychological distress, a proxy for PCMDs, among mothers.

## 2 Methods

### 2.1 Setting

Data for the study were drawn from a large cluster-randomised controlled trial (cRCT) conducted in 60 rural clusters in Dhanusha district in the central Terai region of Nepal. The unit of clustering was the village development committee – the lowest administrative unit in Nepal. The trial protocol has been published and analyses for the main outcomes are being finalised (Shrestha et al., 2011). The trial evaluated two community-based interventions—participatory women's groups focused on maternal and newborn health, and a sepsis management intervention involving training community health workers to treat neonates with sepsis, through a factorial design. For both interventions the primary outcome was neonatal mortality. Additional outcomes included maternal mortality and postnatal psychological distress.

The estimated population of Dhanusha is 754,777, and the 60 clusters had an average population of 6898. Most of the district population is Hindu (90.1%), but there is a relatively large Muslim population (8.4%) (Central Bureau of Statistics, 2008). The four largest caste/ethnic groups in Dhanusha are Yadav (17.6%), Muslim (8.5%), Kewat (6.2%) and Teli (5.0%) (Central Bureau of Statistics, 2001). Most of the population is Madheshi, meaning that they are of plains (Terai) ethnicity, and only a minority is Pahadi (from the hill region). Socioeconomic status and rates of education tend to be lower among Madheshis than Pahadis in the district. Most people live in extended families, and married women live with their husbands' families. Dhanusha is relatively underserved by health facilities: there is approximately one doctor per 23,000 population and one public zonal hospital in Janakpur municipality to serve Dhanusha and five other districts (Shrestha, 2011). Five primary healthcare centres, nine health posts and 88 sub-health posts provide access to public healthcare in rural areas, but a significant proportion of the population consult private doctors (Shrestha, 2011). There are currently no public mental health facilities in Dhanusha, although private psychiatrists hold monthly clinics in Janakpur, the district municipality.

### 2.2 Participants and assessment

During the Dhanusha cRCT, data were collected for mothers residing in intervention and control clusters who delivered between 13th April 2008 and 13th April 2011, however data collection for postnatal distress did not begin until 15th November 2009. In this study we therefore only included data from interviews conducted after this date and until the end of the trial. Deliveries were fully sampled in smaller clusters where fewer than ten deliveries occurred in a single month. In larger clusters, ten women per month were randomly sampled. We excluded mothers temporarily residing in the study clusters to avoid including those who lived outside Dhanusha. We only included data associated with the firstborn infant of a multiple birth, or the first birth during the postnatal distress data collection period if mothers had delivered more than once. There is no consistent definition of the postnatal period in the psychiatric literature, so we adopted a working definition including the first 12 months after delivery, in line with several studies (Miller, 2002; O'Hara, 1988; Rojas et al., 2007). We therefore excluded data associated with mothers who were interviewed after this period.

Women were identified through local informants responsible for identifying all births, and maternal and neonatal deaths, in the study clusters (Shrestha et al., 2011). Interviewers confirmed these events and carried out a structured interview with mothers around six weeks after delivery to collect data on socioeconomic status, perinatal practices, nutrition and health, as well as psychological distress. Data on distress were only collected for livebirths, and not for deliveries associated with a neonatal death or stillbirth. We used the 12-item General Health Questionnaire (GHQ-12) to measure distress. This screening tool has been used to measure common mental disorders, including PCMDs, in a variety of cultural settings (Kuruvilla et al., 1999; Navarro et al., 2007; Patel et al., 2008). The GHQ-12 was validated using a Likert scale in Nepal (Koirala et al., 1999), however to facilitate screening in a largely illiterate population we used a dichotomous scoring approach, which had been validated in a primary care setting in India (Patel et al., 2008). Each of the GHQ-12 items was scored 1 or 0 and a maximum score of 12 was obtainable for each participant. We selected potential risk factors on the basis of previous literature, and using data collected during a qualitative study conducted in the same population (to be published), but were constrained to the factors measured in the Dhanusha cRCT. The following potential predictors of postnatal psychological distress were selected:

*Social and economic factors*: maternal age; caste; ethnicity; asset score (based on the first component of a principal components analysis of variables including land ownership, possession of a mobile phone, television, motor cycle and toilet); maternal education; husband's education; religion; and food security measured using the House and Food Insecurity Access Scale (Coates et al., 2007).

*Gender-based and cultural factors*: age at marriage; having never had a son; ate a restricted diet (excluding carbohydrate constituents) in the first six to seven days after childbirth; and main home during the early postnatal period.

*Reproductive health factors*: parity; past use of temporary family planning methods; serious perinatal health problems (including heavy vaginal bleeding during and before delivery, severe vaginal bleeding after delivery, fits or convulsions during the perinatal period, obstructed labour, retained placenta, high fever, and swollen face, hands and feet); multiple births (i.e. delivery of twins or triplets); level of antenatal care received; non-institutional delivery; caesarean section; short birth spacing (delivered a baby within approximately 33 months of a previous delivery) (World Health Organisation, 2005); and a history of miscarriage, stillbirth, or infant death with previous pregnancies.

### 2.3 Ethical issues

The Dhanusha cRCT received ethical approval from the Nepal Health Research Council and the ethics committee of the Institute of Child Health and Great Ormond Street Hospital for Children, UK. We sought verbal, as opposed to written, informed consent from participants because most of them were illiterate. Women with a GHQ-12 score ≥6 were provided with information about a monthly NGO-funded mental health clinic in Janakpur.

### 2.4 Data analysis

Although we analysed the GHQ-12 data as a continuous outcome, we selected a threshold score of ≥6 to report prevalence of distress, based on the optimum score identified by a study conducted among primary care attenders in Goa (sensitivity 73%, specificity of 90%) (Patel et al., 2008). The intracluster correlation coefficient (ICC) for distress was calculated using a large one-way ANOVA.

**Fig. 1:**
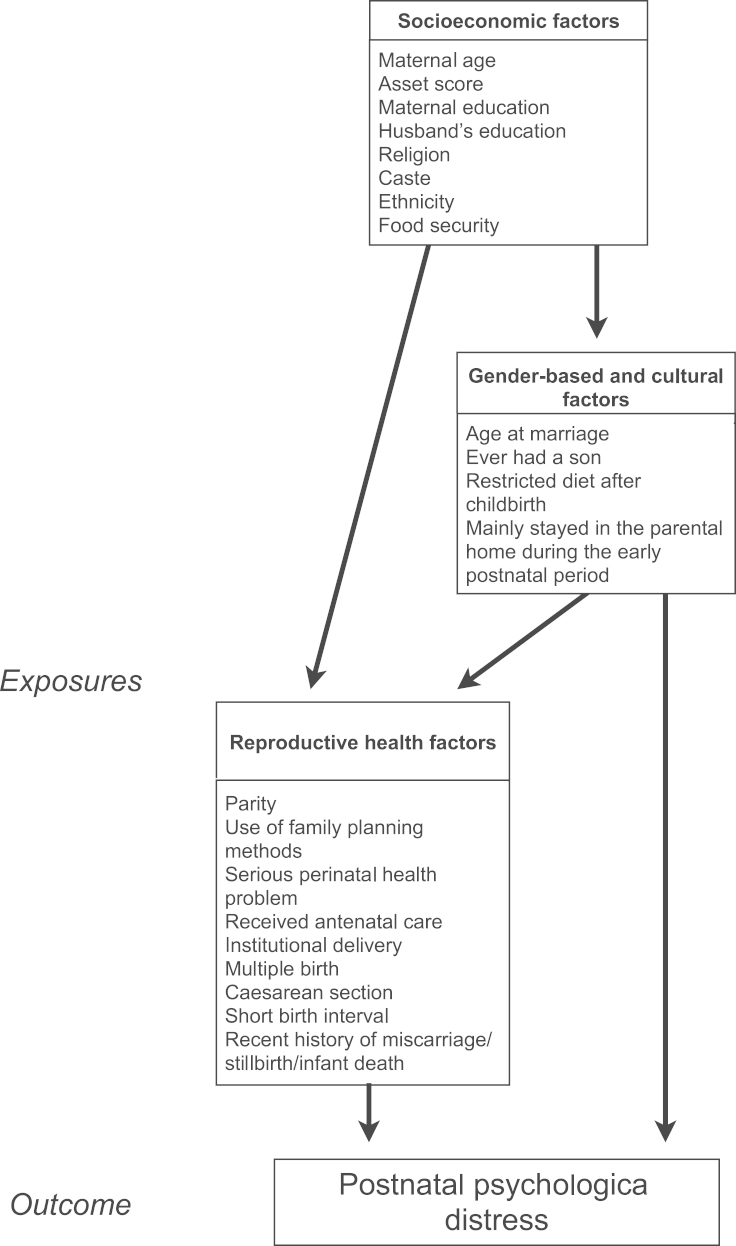
Analytical framework of predictor variables for postnatal psychological distress.

Analyses of risk factors were conducted in two stages. First, the association of each variable with distress was assessed through univariable linear regression models. Second, variables that showed an association at *p*≤0.1 in the first stage were included in multivariable analyses. To guide these multivariable analyses we used an analytical framework based on hierarchical relationships of factors with PCMDs (Fig. 1) (Victora et al., 1997). This approach enabled us to take account of hierarchical inter-relationships between predictors, and to avoid over-reliance on statistical associations (Victora et al., 1997).

We arranged potential risk factors into three levels: the top level consisted of social and economic factors, which we considered to act directly or indirectly through intermediate factors to cause distress; the next level comprised intermediate gender-based and cultural factors; the lowest level included reproductive health factors as the most proximal predictors. According to the framework, groups of variables were entered in hierarchical order into a multivariable modelling procedure. Similar approaches have been used in previous studies to assess predictors of common mental disorders, including PCMDs (Fleck, 2005; Shidhaye and Patel, 2010; Tannous et al., 2008).

### 2.5 Modelling procedure

Univariable analyses tested the association of each variable with GHQ-12 score. The social and economic variables that showed a significant association with GHQ-12 score at the *p*≤0.1 level were included in Model 1. Model 2 included the social and economic variables that remained associated with GHQ-12 score (*p*≤0.1) in Model 1, and gender-based and cultural variables that were associated with the outcome from univariable analyses. In Model 3, variables that were associated with GHQ-12 score in Model 2 (*p*≤0.1) were included along with reproductive health variables that showed association with the outcome in univariable analyses. In Model 4 we used backwards selection of variables to refine the model to include only variables that made a significant contribution (*p*≤0.1). We included maternal age, caste and asset score variables in all multivariable models because there was strong evidence for the association of these factors with mental health (Fisher et al., 2012; Kohrt, 2009; Shidhaye and Patel, 2010; United Nations, 2012). We accounted for clustering of participant characteristics and GHQ-12 score by using generalised estimating equation regression models (of the Gaussian family using the identity link) in univariable and multivariable analyses. We included variables in the models to control for allocation to women's group and the sepsis management intervention because the interventions may have affected predictors of psychological distress, such as neonatal mortality, and maternal and infant health problems (Tripathy et al., 2010). We applied probability weights to the data to reflect the differential sampling strategies for large versus small clusters, and adjusted for stratified randomisation of clusters since clusters were randomised to intervention and control arms on the basis of cluster population size (Shrestha et al., 2011). The GHQ-12 data were log transformed to reduce data skew and improve the fit of the regression model. Before conducting the log transformation we added 0.5 to each value to account for the zeros in the dataset. We transformed the results back to the original scale and these are interpreted as the ratio of the geometric mean GHQ-12 score for the variable category over the geometric mean GHQ-12 score for the reference category. The limits of the confidence interval are the minimum and maximum ratios, and significant results are those that do not span 1. We carried out casewise deletion of participants with missing outcome and predictors data because rates of missing data were relatively low and exclusion of these cases did not compromise power and precision of the results. All analyses were carried out in Stata version 12 (College Station, TX: StataCorp LP).

## 3 Results

**Fig. 2:**
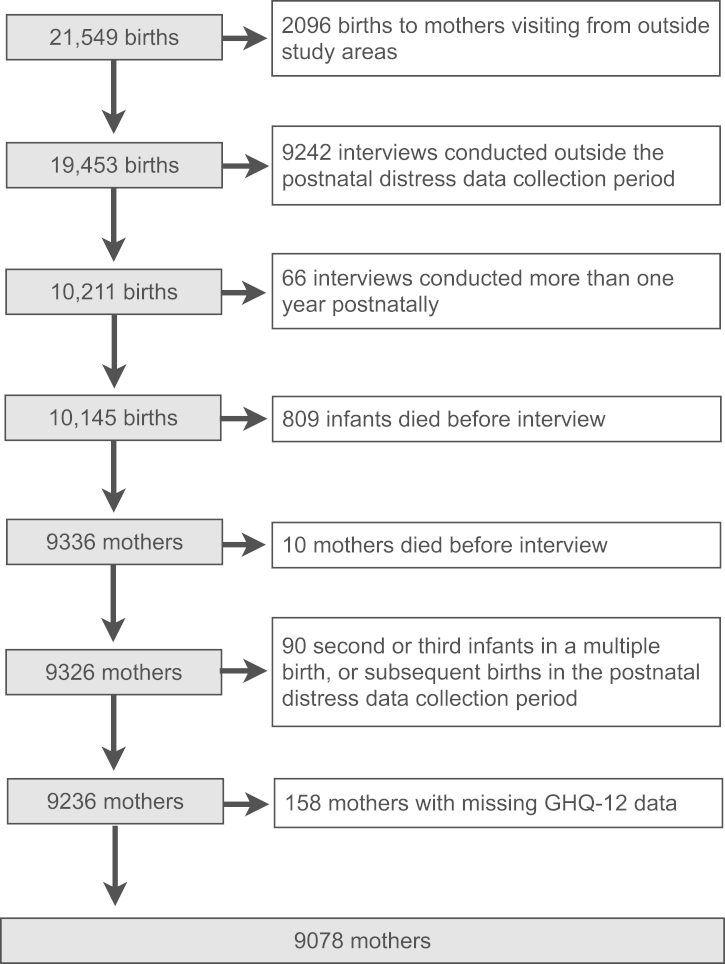
Flow chart outlining the sample selection procedure for the analysis of risk factors for postnatal psychological distress.

Fig. 2 describes the selection of the sample for analysis. Data were available for 21,549 mothers who gave birth between 13th April 2008 and 13th April 2011. We excluded data from 2096 mothers who were temporary residents in the study area, 9242 mothers interviewed outside the postnatal distress data collection period, and 66 mothers interviewed more than one year postnatally. We removed data associated with 809 neonatal deaths, 10 maternal deaths and 90 births to mothers who had already delivered during the postnatal distress data collection period. A further 157 mothers with missing GHQ-12 data were excluded (casewise deletion). In total, 9078 mothers were included in the final sample. The median interval between birth and interview was 41 days (interquartile range 32–55 days).

### 3.1 Participant characteristics

Table 1 describes key characteristics of participants. The mean age of the sample was 24 years (SD 4.9). The majority of mothers were Hindu (89%) and Madheshi (96%); 20% and 18% belonged to Yadav and Dalit castes respectively. Over two thirds (72%) of mothers had received no schooling, compared to 45% of their husbands. Almost one third of mothers were married before they were 16, and one third were primiparous. Nearly a third received no antenatal care and only 26% delivered in an institution. At 902 female infants per 1000 male infants, the sex ratio at birth was unbalanced.

### 3.2 Prevalence of and risk factors for postnatal psychological distress

The prevalence of postnatal psychological distress in the study population (GHQ-12 score ≥6) was 9.8% (886/9078). GHQ-12 scores were highly clustered (ICC 0.18 (0.12, 0.23).

Table 2 reports results of univariable analyses exploring the association of social and economic, gender-based, cultural, and reproductive health factors, with GHQ-12 score. Among social and economic factors, asset score, caste, maternal education, husband's education, ethnicity and food security were associated with GHQ-12 score with *p*≤0.1; religion and maternal age were not. Among the gender-based and cultural factors tested, participants who had never had a son and who did not stay with their parents in the early postnatal period had significantly higher GHQ-12 scores. By contrast, there was no association between GHQ-12 score and variables for age at marriage and having a restricted diet after childbirth. The following reproductive health factors were associated with GHQ-12 score: parity, serious perinatal health problems, multiple birth, antenatal care received, institutional delivery, and caesarean section. Having a short birth interval, use of family planning methods and a recent history of miscarriage, stillbirth or infant death, were not associated with GHQ-12 score. Overall, the largest effects were observed for severe food insecurity and having had a multiple birth.

Table 3 shows results of systematic multivariable analyses of factors that were associated with GHQ-12 score in univariable analyses. In Model 1, a lower asset score, having no schooling or a husband with no schooling, and food insecurity retained an independent association with GHQ-12 score. The largest association was found with severe food insecurity. Maternal age, caste and ethnicity were not associated with GHQ-12 score.

In Model 2, a lower asset score, having no schooling or a husband with no schooling, food insecurity, having never had a son and not staying in the parental home in the postnatal period, were independently associated with GHQ-12 score. A lower asset score, no schooling or having a husband with no schooling, food insecurity, having never had a son and not staying in the parental home in the postnatal period were also associated with GHQ-12 score in Model 3, as was lower maternal age, higher parity, having a serious perinatal health problem, multiple birth, caesarean section, and poor or no antenatal care. In the final model (Model 4), after backwards selection of variables resulting in the removal of the variable for institutional delivery, factors independently associated with GHQ-12 score were: severe food insecurity (*β* 2.21 (1.43, 3.40) *p*<0.001), multiple birth (*β* 2.28 (1.27, 4.10) *p*=0.006), caesarean section (*β* 1.70 (0.29, 2.24) *p*=0.053), serious perinatal health problems (*β* 1.58 (1.23, 2.02) *p*<0.001), no schooling (*β* 1.37 (1.08, 1.73) *p*=0.010), lower asset score (*β* 1.33 (1.10, 1.60) *p*=0.003), higher parity (*β* 1.33 (1.09, 1.61) *p*=0.005), poor or no antenatal care (*β* 1.31 (1.15, 1.48) *p*<0.001), having never had a son (*β* 1.31 (1.14, 1.49) *p*<0.001), not staying in the parental home in the postnatal period (*β* 1.15 (1.02, 1.30) *p*=0.021), having a husband with no schooling (*β* 1.17 (0.96, 1.43)) *p*=0.115) and lower maternal age (*β* 0.99 (0.97, 1.00) *p*=0.043).

## 4 Discussion

Our findings confirm the importance of established predictors of PCMDs (fewer assets, no schooling, lower maternal age, higher parity, having never had a son, perinatal health problems, poor or no antenatal care and caesarean section), and identify new predictors (food insecurity, having a multiple birth and not staying in the parental home in the postnatal period) in a rural population in Nepal. Using a GHQ-12 threshold score of ≥6, our estimate of the prevalence of distress (9.8%) falls within the range of estimates reported in previous studies with postnatal women in Nepal (3% to 12%) (Ho-Yen et al., 2006; Nepal et al., 1999; Regmi et al., 2002). We found that GHQ-12 scores were highly clustered (ICC 0.18 (0.12, 0.23), possibly due to cluster-level variability in predictors of distress. Other studies conducted in South Asia have reported lower ICCs, ranging from 0.08 using the Kessler 10 item scale, to 0.05 using the structured clinical interview for Diagnostic and Statistical Manual of Mental Disorders (DSM)–IV diagnosis (Prost et al., 2012; Rahman et al., 2008). Research is needed to identify community-level predictors of distress and how these impact on individual experiences of mental ill health.

The study has several limitations. We tested multiple variables with the chance of incurring type I and type II errors. However, we mitigated this by selecting variables based on prior research, using univariable analyses to explore relationships between variables and outcome, and making an a priori decision to retain maternal age, caste and asset score in the models (Grobbee and Hoes, 2009; Sun et al., 1996). We did not formulate or test hypotheses about interactions between predictors, though it is likely that such interactions exist. In addition, because of the cross-sectional study design we were unable to establish causality. Data were not collected on several known predictors of PCMDs, including infant death, domestic violence and psychiatric history. We were therefore unable to assess the association of these factors with distress. Finally, some women may have been distressed during pregnancy, as well as postnatally, and therefore predictors identified in this study may also be relevant for antenatal common mental disorders.

### 4.1 Reproductive health is crucial for maternal mental health

We found that mothers who received inadequate or no antenatal healthcare, or had a serious perinatal health problem, caesarean section or multiple birth, were at risk of postnatal psychological distress. Studies have reported links between poor reproductive health and PCMDs in other low and middle-income settings, as well as high-income settings (Fisher et al., 2012; Prost et al., 2012). Improving reproductive health is already a priority in low-income countries since rates of maternal mortality and morbidity are elevated (Hogan et al., 2010). User fees can be a barrier to accessing adequate healthcare in settings where the private health sector predominates, such as Nepal (van Doorslaer et al., 2006). Paying for emergency or operative procedures such as caesarean section may push families into poverty and debt, giving rise to further psychological distress (Filippi et al., 2006; Patel et al., 1999). Consequently, interventions that increase access to antenatal care and rates of deliveries attended by skilled birth assistants, and improve perinatal care practices, are likely to reduce the burden of PCMDs. There have been recent calls to integrate mental health initiatives into existing maternal and child health programmes (Rahman, 2013). Suicide is the leading cause of death among women of reproductive age in Nepal, and since suicide is associated with common mental disorders, such integrative approaches may be beneficial in this setting (Harris and Barraclough, 1997; Suvedi et al., 2009).

We found that short birth intervals and use of family planning methods were not associated with GHQ-12 score. Studies in Nepal and South Asia have described how women's status is linked to their fertility: women without children assume the lowest status in the family hierarchy and are considered to be ‘incomplete’ (Winkvist and Akhtar, 2000). A qualitative study conducted in northwestern Nepal found that women were pressured to bear many children to increase their labour resources (Kohrt et al., 2009). However, that women with five or more children were at increased risk of distress suggests a limit to any beneficial effects of having a large family.

### 4.2 Understanding cultural factors

We found no association between age at marriage and postnatal psychological distress, though the data suggest that early marriage is a problem in Dhanusha: 30% (2704/9078) of participants were married before the age of 16 and 181 (2%) were married under the age of 13. Other studies have also reported a high rate of early marriage in the Terai (Choe et al., 2004; Ministry of Health and Population et al., 2012). Women who are married at a young age may be more vulnerable because they are less educated and because early marriage is associated with early motherhood and an increased risk of perinatal health problems (Choe et al., 2004; Kongnyuy et al., 2008; Sagili et al., 2012). Married women are expected to live with their husbands' families, and girls who are separated from their own families at a young age might feel more distressed (Bennett, 1983). Two possible explanations for the null association between age at marriage and distress are that: (i) although women are married at an early age, cohabitation of married couples is often delayed until the woman is older; (ii) marriage in Dhanusha is a livelihood strategy for women, and therefore confers a protective effect. Qualitative work is required to understand experiences of early marriage in this population.

Although most women remained in their marital homes after delivery, 17% (1072/6206) of multiparas, and 34% (954/2826) of primiparas spent most of the early postnatal period in their parents' home. These women may have been at a reduced risk of distress because they were relieved of the domestic responsibilities of a daughter in-law, and were able to recover in the care of their mothers and sisters.

### 4.3 Socioeconomic and gender disadvantage predict postnatal psychological distress: advocating a social determinants perspective on maternal mental health

In contrast to previous studies in Nepal, we did not find an independent association between caste and postnatal distress, possibly because potential mediating variables such as asset score and food insecurity were included in the multivariable regression models (Kohrt, 2009; Kohrt et al., 2012, 2009). This, and the fact that most participants in the study were Madheshi, may explain the absence of an independent association between Madheshi ethnicity and distress.

Our data show that other poverty-related variables – having a lower asset score, no schooling, a husband with no schooling, and food insecurity – were associated with postnatal distress and are consistent with previous meta-analyses of predictors of common mental disorders (Fisher et al., 2012; Lund et al., 2010). Although we cannot exclude the possibility that people with mental health problems are more likely to drift into poverty, poverty may lead to psychological distress through social exclusion, increased exposure to crime and violence and increased risk of disease as a result of more risky health behaviours and inability to afford adequate healthcare and nutrition (Lund et al., 2010; Murali and Oyebode, 2004). Food insecurity may lead to anxiety about accessing adequate food or force individuals to acquire food in socially unacceptable ways (Weaver and Hadley, 2009). Undernutrition may also have a direct effect on mental health (Hoek et al., 1996; Venables and Raine, 2012).

Education may be protective for mental health by providing individuals with knowledge to inform attitudes towards lifestyle and health behaviours (Brown et al., 1986; LeVine et al., 2004; Patel et al., 1999). Better educated women choose to marry later, are more likely to be involved in family planning, earn more and exercise greater control over household resources, which may lead to better mental health (Qadir et al., 2011). Education may therefore influence aspirations, self-image and confidence. Lack of education may be a manifestation of gender disadvantage in contexts where sons are preferentially sent to school, while daughters remain in the home. We also showed that mothers who have never had a son are vulnerable to psychological distress. In Nepal, gender disadvantage is rooted in religion and a traditionally patrilineal society. Son preference is linked to poverty, and to the absence of a welfare system, meaning that sons are required to provide financial security for their parents in later life (Bennett, 1983). Women therefore experience pressure from family and the community to have sons, and those who have never had sons may experience hostility.

In conclusion, our data support recent calls for interventions to address societal risk factors such as poverty, education and gender disadvantage, with a focus on long term prevention to reduce the burden of mental illness at a population level (Lund et al., 2010; Clarke et al., 2013). To achieve this, inclusion of mental health on development agendas and collaboration between health and development sectors is necessary (Lund et al., 2010; Miranda and Patel, 2005).

## 5 Conclusions

Poor perinatal health, and socioeconomic and gender disadvantage, are key predictors of PCMDs in Dhanusha, Nepal. Integration of mental health into existing maternal and child health programmes, as well as collaboration between health and development partners to tackle poverty and access to education, are likely to improve maternal mental health in this setting.

## Role of funding source

Funding for the trial from which our data were sourced was provided by UBS Optimus Foundation, United States Agency for International Development, a “Population Science of Maternal and Child Survival” Wellcome Trust Strategic Award, and the UK Department for International Development Towards 4+5 Research Programme Consortium. Kelly Clarke was supported by a Medical Research Council PhD studentship. The funders had no role in the design of the study, data collection and analysis, interpretation of the findings or writing of the paper. The corresponding author had access to all the data and had final responsibility for the decision to submit for publication.

## Conflict of interest

We declare that we have no conflict of interest in the authorship or publication of this article.

## Figures and Tables

**Table 1:** Characteristics of mothers included in the study.

	***N***	**%**
**Age (*****N*****=9076)**		
Mean (SD)	23.8	4.9
Median (range)	23	(14–48)

**Caste (*****N*****=9078)**		
Brahmin	143	1.6
Yadav	1777	19.6
Koiri/Sudi/Teli	1472	16.2
Janajati	1607	17.7
Mandal	1425	15.7
Muslim	991	10.9
Dalit	1630	18.0
Other	33	0.4

**Religion (*****N*****=9074)**		
Hindu	8097	89.2
Muslim	959	10.6
Buddhist	15	0.2
Other	3	0.0

**Ethnicity (*****N*****=9077)**		
Hills (Pahadi)	354	3.9
Plains (Madheshi)	8723	96.1

**Maternal education (*****N*****=8979)**		
Higher secondary or higher	643	7.2
Secondary	714	8.0
Primary/pre-primary/non-formal	1165	13.0
No schooling	6457	71.9

**Husband's education (*****N*****=9007)**		
Higher secondary or higher	1689	18.8
Secondary	1397	15.5
Primary/pre-primary/non-formal	1884	20.9
No schooling	4037	44.8

**Age at marriage (*****N*****=9078)**		
18+	1340	14.8
16–17	5034	55.5
13–15	2523	27.8
<13	181	2.0

**Parity (*****N*****=9042)**		
Mean (SD)	2.5	1.4
Median (range)	2	1–11
Primiparity	2829	31.3

**Sex of infant (*****N*****=9076)**		
Male	4771	52.6
Female	4305	47.4

**Antenatal care (*****N*****=9027)**		
Satisfactory care	2011	22.3
Some care	4080	45.2
No care	2936	32.5

**Institutional delivery (*****N*****=8942)**		
Delivered in an institution	2276	25.5

**Table 2:** Univariable analyses of association between candidate determinants and postnatal psychological distress.

	**Frequency (%)**	**Prevalence (%)**	***β*****(95% CI)**	***P*****-value**	**Overall*****P-*****value**
***Socioeconomic factors***					
**Maternal age in years(*****N*****=9076)**					0.551
Mean age (SD)	23.8 (4.9)		1.00 (0.99, 1.02)	0.551	
<20	1810 (19.9)	181 (10.0)			
20–24	3462 (38.1)	333 (9.6)			
25–29	2600 (28.7)	244 (9.4)			
30–34	824 (9.1)	90 (10.9)			
>34	380 (4.2)	886 (9.8)			
**Asset score (first asset quintile: richest,*****N*****=9001)**					<0.001
First	1796 (20.0)	77 (8.7)	[ref]		
Second	1802 (20.0)	83 (9.4)	1.23 (1.07, 1.41)	0.004	
Third	1796 (20.0)	77 (8.7)	1.36 (1.20, 1.56)	<0.001	
Fourth	1793 (19.9)	100 (11.3)	1.46 (1.24, 1.73)	<0.001	
Fifth	1814 (20.2)	110 (12.4)	1.96 (1.63, 2.37)	<0.001	
**Caste (*****N*****=9078)**⁎					<0.001
Advantaged	174 (1.9)	12 (6.9)	[ref]		
Less advantaged	2766 (30.5)	238 (8.6)	1.34 (0.97, 1.83)	0.072	
Not advantaged	4508 (49.7)	458 (10.2)	1.48 (1.07, 2.05)	0.018	
Disadvantaged	1630 (18.0)	178 (10.9)	1.79 (1.33, 2.42)	<0.001	
**Maternal education (*****N*****=8979)**					<0.001
Higher secondary and higher	643 (7.2)	39 (6.1)	[ref]		
Secondary	714 (8.0)	49 (6.9)	1.12 (0.90, 1.38)	0.308	
Primary/preprimary/non-formal	1165 (13.0)	92 (7.9)	1.45 (1.18, 1.77)	<0.001	
No schooling	6457 (71.9)	699 (10.8)	1.85 (1.53, 2.24)	<0.001	
**Husband's education (*****N*****=9007)**					<0.001
Higher secondary and higher	1689 (18.8)	114 (6.8)	[ref]		
Secondary	1397 (15.5)	131 (9.4)	1.15 (1.01, 1.31)	0.041	
Primary/preprimary/non-formal	1884 (20.9)	155 (8.2)	1.19 (1.02, 1.37)	0.022	
No schooling	2037 (44.8)	481 (11.9)	1.74 (1.49, 2.02)	<0.001	
**Religion (*****N*****=9074)**					0.623
Hindu	8097 (89.2)	780 (9.6)	[ref]		
Muslim or other	977 (10.8)	106 (10. 9)	1.03 (0.91, 1.18)	0.623	
**Ethnicity (*****N*****=9077)**					0.013
Hills (Pahadi)	354 (3.9)	14 (4.0)	[ref]		
Plains (Madheshi)	8723 (96.1)	872 (10.0)	1.44 (1.08, 1.93)	0.013	
**Food security (HFIAS scale) (*****N*****=8994)**					<0.001
Food secure	6023 (67.0)	487 (8.1)	[ref]		
Mildly food insecure	1130 (12.6)	118 (10.4)	1.18 (0.94, 1.48)	0.142	
Moderately food insecure	1500 (16.7)	211 (14.1)	1.66 (1.28, 2.16)	<0.001	
Severely food insecure	341 (3.8)	55 (16.1)	2.94 (1.89, 4.55)	<0.001	

***Gender-based and cultural factors***					
**Age at marriage (*****N*****=9078)**					0.333
18+	1340 (14.8)	91 (6.8)	[ref]		
16-17	5034 (55.5)	528 (10.5)	1.18 (1.01, 1.37)	0.034	
13–15	2523 (27.8)	247 (9.8)	1.18 (0.97, 1.43)	0.101	
< 13	181 (2.0)	20 (11.1)	0.98 (0.70, 1.37)	0.906	
**Ever had a son (*****N*****=9047)**					0.003
Yes	6753 (74.6)	609 (9.0)	[ref]		
No	2294 (25.4)	271 (11.8)	1.25 (1.08, 1.44)	0.003	
**Restricted diet after childbirth (*****N*****=9070)**					0.504
No	7576 (83.5)	753 (9.9)	[ref]		
Yes	1494 (16.5)	132 (8.8)	0.92 (0.73, 1.17)	0.504	
**Mainly stayed in the parental home for the early postnatal period (*****N*****=9064)**					0.044
Yes	2032 (22.4)	210 (10.3)	[ref]		
No	7032 (77.6)	676 (9.6)	1.14 (1.00, 1.29)	0.044	

***Reproductive health factors***					
**Parity (*****N*****=9042)**					0.022
1-2 children	5282 (58.4)	469 (8.9)	[ref]		
3-4 children	2936 (32.5)	295 (10.1)	1.18 (0.90, 1.14)	0.558	
5 or more children	824 (9.1)	119 (14.4)	1.34 (0.09, 1.66)	0.006	
**Use of family planning methods (*****N*****=9074)**					0.840
Yes	226 (2.5)	24 (10.6)	[ref]		
No	8848 (97.5)	862 (9.7)	1.02 (0.80, 1.31)	0.840	
**Serious perinatal health problem (*****N*****=9078)**					<0.001
No	8301 (91.4)	756 (9.1)	[ref]		
Yes	777 (8.6)	130 (16.7)	1.68 (1.30, 2.19)	<0.001	
**Multiple birth (*****N*****=9076)**					0.005
Singleton	9014 (99.3)	874 (9.7)	[ref]		
Twins or triplets	62 (0.7)	12 (19.4)	2.31 (1.29, 4.15)	0.005	
**Antenatal care received (*****N*****=9027)**					<0.001
Satisfactory care	2011 (22.3)	144 (7.2)	[ref]		
Some care	4080 (45.2)	383 (9.4)	1.30 (1.15, 1.46)	<0.001	
No care	2936 (32.5)	357 (12.2)	1.53 (1.35, 1.73)	<0.001	
**Institutional delivery (*****N*****=8942)**					0.015
Yes	2276 (25.5)	228 (10.0)	[ref]		
No	6666 (74.6)	649 (9.7)	1.15 (1.03, 1.30)	0.015	
**Caesarean section (*****N*****=9078)**					0.001
No	8698 (95.8)	822 (9.5)	[ref]		
Yes	380 (4.2)	64 (16.8)	1.67 (1.24, 2.26)	0.001	
**Short birth interval (*****N*****=9031)**					0.406
No	6654 (73.7)	671 (10.1)	[ref]		
Yes	2377 (26.3)	213 (9.0)	0.96 (0.87, 1.06)	0.406	
**Recent history of miscarriage/stillbirth /infant death (*****N*****=9029)**					0.223
No (infant is alive or no previous pregnancies)	8598 (95.2)	837 (9.7)	[ref]		
Yes	431 (4.8)	47 (10.9)	1.18 (0.90, 1.54)	0.223	

[ref] reference category.

⁎ Caste categories: disadvantaged low castes—Dalit; non-advantaged—Janajati, Mandal and Muslim groups; advantaged high and middle caste groups—Brahman, Yadav, Koiri and Sudi/Teli.

**Table 3:** Multivariable analyses of association between candidate determinants and postnatal psychological distress.

		**Model 1**			**Model 2**			**Model 3**			**Model 4**		
		***β*****(95% CI)**	***p***	**Overall*****p***	***β*****(95% CI)**	***p***	**Overall*****p***	***β*****(95% CI)**	***p***	**Overall*****p***	***β*****(95% CI)**	***p***	**Overall*****p***
***Socioeconomic factors***													
**Maternal age, years**													
		1.00 (0.98, 1.01)	0.510	0.598	1.00 (0.99, 1.01)	0.916	0.959	0.99 (0.97, 1.00)	0.036	0.050	**0.99 (0.97, 1.00)**	**0.030**	**0.043**
**Asset score (first asset quintile: richest)**				0.008			0.009			0.028			**0.020**
First		[ref]			[ref]			[ref]			**[ref]**		
Second		1.14 (0.99, 1.31)	0.070		1.14 (1.00, 1.31)	0.059		1.14 (0.99, 1.31)	0.061		**1.14 (0.99, 1.30)**	**0.069**	
Third		1.19 (1.04, 1.36)	0.010		1.19 (1.04, 1.36)	0.011		1.20 (1.04, 1.37)	0.011		**1.20 (1.05, 1.38)**	**0.007**	
Fourth		1.16 (0.98, 1.37)	0.086		1.17 (0.99, 1.38)	0.068		1.14 (0.96, 1.35)	0.145		**1.15 (0.97, 1.36)**	**0.113**	
Fifth		1.34 (1.12, 1.60)	0.002		1.35 (1.12, 1.61)	0.001		1.32 (1.10, 1.59)	0.003		**1.33 (1.10, 1.60)**	**0.003**	
**Caste**⁎				0.855			0.981			0.736			**0.770**
Advantaged		[ref]			[ref]			[ref]			**[ref]**		
Less advantaged		0.96 (0.65, 1.42)	0.842		1.16 (0.86, 1.58)	0.337		1.21 (0.90, 1.62)	0.199		**1.20 (0.90, 1.61)**	**0.206**	
Not advantaged		0.97 (0.66, 1.44)	0.896		1.16 (0.83, 1.63)	0.388		1.21 (0.88, 1.66)	0.246		**1.21 (0.88, 1.66)**	**0.248**	
Disadvantaged		0.94 (0.64, 1.39)	0.757		1.13 (0.82, 1.55)	0.469		1.19 (0.89, 1.61)	0.245		**1.19 (0.88, 1.60)**	**0.268**	
**Maternal education**				0.007			0.008			0.030			**0.026**
Higher secondary and above		[ref]			[ref]			[ref]			**[ref]**		
Secondary		1.06 (0.86, 1.32)	0.576		1.07 (0.87, 1.32)	0.516		1.10 (0.88, 1.36)	0.412		**1.08 (0.87, 1.33)**	**0.505**	
Primary/pre-primary/ non-formal		1.32 (1.07, 1.64)	0.011		1.32 (1.07, 1.64)	0.011		1.32 (1.06, 1.66)	0.015		**1.31 (1.05, 1.63)**	**0.018**	
No schooling		1.41 (1.13, 1.75)	0.002		1.42 (1.14, 1.78)	0.002		1.37 (1.08, 1.75)	0.010		**1.37 (1.08, 1.73)**	**0.010**	
**Husband's education**				0.069			0.056			0.098			**0.079**
Higher secondary and higher		[ref]			[ref]			[ref]			**[ref]**		
Secondary		0.98 (0.85, 1.12)	0.738		0.97 (0.84, 1.11)	0.629		0.96 (0.83, 1.12)	0.633		**0.98 (0.85, 1.13)**	**0.796**	
Primary/pre-primary/ non-formal		0.91 (0.80, 1.04)	0.175		0.90 (0.79, 1.03)	0.132		0.90 (0.78, 1.03)	0.112		**0.90 (0.78, 1.03)**	**0.111**	
No schooling		1.18 (0.97, 1.45)	0.094		1.19 (0.97, 1.45)	0.092		1.16 (0.95, 1.42)	0.148		**1.17 (0.96, 1.43)**	**0.115**	
**Ethnicity**				0.101									
Hills (Pahadi)		[ref]											
Plains (Madheshi)		1.27 (0.91, 1.77)	0.156										
**Food security (HFIAS scale)**				<0.001			0.001			0.001			**0.001**
Food secure		[ref]			[ref]			[ref]			**[ref]**		
Mildly food insecure		1.01 (0.80, 1.28)	0.906		1.01 (0.80, 1.28)	0.936		1.01 (0.79, 1.28)	0.965		**1.01 (0.80, 1.29)**	**0.923**	
Moderately food insecure		1.36 (1.05, 1.76)	0.021		1.36 (1.05, 1.76)	0.021		1.33 (1.02, 1.73)	0.035		**1.36 (1.04, 1.77)**	**0.023**	
Severely food insecure		2.25 (1.45, 3.48)	<0.001		2.23 (1.44, 3.46)	<0.001		2.19 (1.42, 3.39)	<0.001		**2.21 (1.43, 3.40)**	<**0.001**	

***Gender-based and cultural factors***													
**Ever had a son**							<0.001			<0.001			<**0.001**
Yes					[ref]			[ref]			**[ref]**		
No					1.31 (1.14, 1.49)	<0.001		1.31 (1.14, 1.49)	<0.001		**1.31 (1.14, 1.49)**	<**0.001**	
**Mainly stayed in the parental home for the early postnatal period**							0.026			0.039			**0.034**
Yes					[ref]			[ref]			**[ref]**		
No					1.16 (1.03, 1.31)	0.017		1.15 (1.02, 1.30)	0.024		**1.15 (1.02, 1.30)**	**0.021**	

***Reproductive health factors***													
**Parity**													
1-2 children								[ref]		0.010	**[ref]**		**0.009**
3-4 children								1.09 (0.97, 1.23)	0.148		**1.09 (0.97, 1.22)**	**0.146**	
5 or more children								1.32 (1.09, 1.61)	0.005		**1.33 (1.09, 1.61)**	**0.005**	
**Serious perinatal health problem**										<0.001			<**0.001**
No								[ref]			**[ref]**		
Yes								1.59 (1.23, 2.05)	<0.001		**1.58 (1.23, 2.02)**	<**0.001**	
**Multiple birth**										0.005		**0.006**	**0.006**
Singleton								[ref]			**[ref]**		
Twins or triplets								2.36 (1.31, 4.27)	0.004		**2.28 (1.27, 4.10)**		
**Antenatal care received**										<0.000			<**0.001**
Satisfactory care								[ref]			**[ref]**		
Some care								1.21 (1.06, 1.38)	0.004		**1.21 (1.06, 1.38)**	**0.004**	
No care								1.30 (1.13, 1.49)	<0.001		**1.31 (1.15, 1.48)**	<**0.001**	
**Institutional delivery**										0.552			
Yes								[ref]					
No								1.05 (0.92, 1.20)	0.459				
**Caesarean section**										<0.001			<**0.001**
No								[ref]			**[ref]**		
Yes								1.71 (1.30, 2.26)	<0.001		**1.70 (1.29, 2.24)**	<**0.001**	

[ref] reference category.

⁎ Caste categories: disadvantaged low castes—Dalit; non-advantaged—Janajati, Mandal and Muslim groups; advantaged high and middle caste groups—Brahman, Yadav, Koiri and Sudi/Teli.
